# Giant Cell Tumor of the Carotid Body: A Rare Tumor with Malignant Potential

**DOI:** 10.22038/IJORL.2022.58598.3027

**Published:** 2022-07

**Authors:** Rocio Bruballa, Luis Boccalatte, Ana Jaen, Marcelo Figari, Juan Jose Larranaga

**Affiliations:** 1 *General Surgery Service, Hospital Italiano de Buenos Aires, Juan D. Perón 4190, C1199ABD Buenos Aires, Argentina.*; 2 *Department of Head and Neck Surgery, Hospital Italiano de Buenos Aires, Juan D. Perón 4190, C1199ABD Buenos Aires, Argentina.*; 3 *Pathology Service, Hospital Italiano de Buenos Aires, Juan D. Perón 4190, C1199ABD Buenos Aires, Argentina.*; 4 *Department of Plastic and Reconstructive Surgery. Hospital Italiano de Buenos Aires, Juan D. Perón 4190, C1199ABD Buenos Aires, Argentina.*

**Keywords:** Carotid body tumors, Carotid angiography, Malignant findings, Internal carotid artery ligation

## Abstract

**Introduction:**

Carotid body tumors (CBTs) are certainly unusual. They are vascular lesions originating from paraganglionic cells, located at the common carotid artery (CCA) bifurcation. They represent less than 0.5% of head and neck tumors, approximately 1-3 cases per million. Malignant CBTs are extremely rare; in the literature, published rates on average are < 10%. The diagnostic criteria for malignancy should be based on the finding of distant metastasis. Due to its unpredictable nature and its malignant potential, diagnosis before metastasis and complete surgical resection are the keys to a favorable prognosis.

**Case Report:**

Given little experience in CBTs, its biology and treatment remain uncertain. We present the case of a 48-years-old patient, with a mass on the left side of the neck that was found to be a vast CBT with suspicious histopathology. Its size, rare location, pathologic findings, and management strategy applied for its treatment, illustrate an unusual case that highlights the importance of its publication.

**Conclusions:**

CBT is rare, but subject to cure lesion if resected without metastatic or residual disease. This is why surgery should be performed whenever possible and why it is so necessary to study this pathology thoroughly and to take it into account in the differential diagnosis.

## Introduction

The carotid body is a small structure, located in the adventitia of the CCA bifurcation. Part of the sympathetic nervous system, is composed of chromaffin-negative glomus cells and works as a vascular chemoreceptor. CBTs are a rare group of non-functional, slow-growing, mostly benign tumors, originating from paraganglionic cells of the carotid body (1). They represent less than 0.5% of head and neck tumors, approximately 1-3 cases per million inhabitants per year (2). A surgical classification system was developed by Shamblin et al. (3) to predict surgical morbidity. It describes the extent to which the CBT envelopes the CCA, the internal carotid artery (ICA), and the external carotid artery (ECA) and was designed to be a predictor of intraoperative technical difficulty.

Shamblin III tumors or “Giant” CBTs are extremely rare and management of such highly vascular lesions could represent a complex challenge (4,5). Malignant CBTs are extremely unusual: published rates among existing literature range between 6-30%, mostly averaging <10% (6). Given the little experience in CBTs, its biology and treatment remain uncertain. We present the case of a 48-years-old patient, with a mass on the left side of the neck that was found to be a vast CBT with suspicious histopathology. Its size, rare location, pathologic findings, and management strategy applied for its treatment, illustrate an unusual case that highlights the importance of its publication.

## Case Report

**Fig 1:**
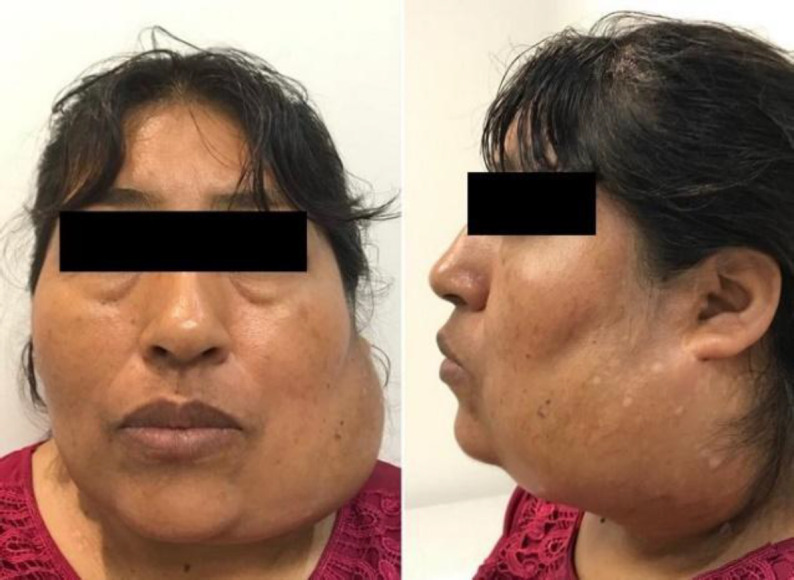
Patient's preoperative photograph

A 48-year-old female patient, from Bolivia (Fig 1), consulted at our hospital for a painful, fixed, firm lesion, on the left side of her neck.

**Fig 2:**
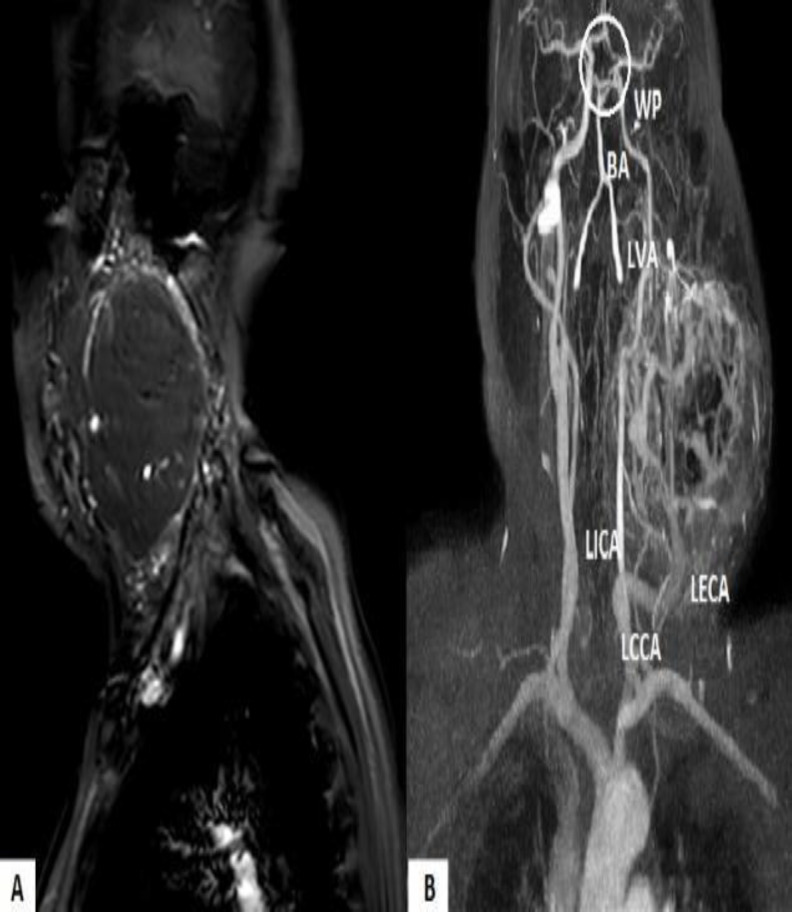
(A) Sagittal AngioMRI image showing mass lesion in the left carotid space displacing vascular structures. (B) Coronary AngioMRI image showing intensely hyperdense mass lesion, in the left carotid space pushing the left internal carotid artery (LICA). The lesion is supplied by the external carotid artery (LECA) and its branches. Willis polygon (WP); Basilar Artery (BA); Common carotid artery (CCA)

A Magnetic Resonance Image (MRI) was requested to evaluate the extent of the lesion and its anatomic characteristics, to allow staging and surgical planning. It showed a voluminous expansive lesion centered in the left carotid space, compatible with paraganglioma of the carotid body with a maximum diameter of 100 mm (Fig 2).

The left ICA, the ECA, as well as the left internal jugular vein (IJV) were displaced dorsally and compressed at the level of the proximal and middle section of the cervical segment (C1), with an absence of flow signal. The study also showed an alteration in the intracranial flow signal, linked to slow/absent antegrade flow due to the cervical compression of the ICA described above, possibly associated with arterial circle substitution. 

**Fig 3:**
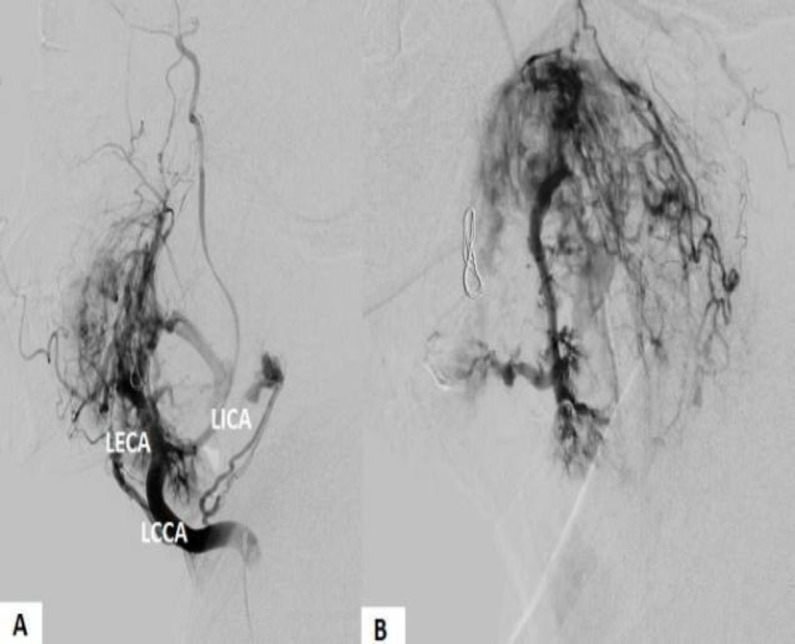
(A-B) Carotid angiography revealing a carotid body tumor irrigated by the left external carotid artery (LECA)

To clarify the case, a carotid angiography (Fig 3) was performed. A tumor irrigated by the ECA was observed, which compressed the ICA, causing severe stenosis. The IJV was also completely occluded. No metastasis was reported.

To accurately predict the possible consequences of direct ICA ligation, a balloon occlusion test (BOT) was carried out. The patient tolerated the study adequately, without developing a neurologic deficit. There were no manifestations of hypo perfusion after measuring the cerebral blood flow, cerebral blood volume, and mean transit time. These results showed that an ICA sacrifice without the need for bypass could be performed. 

The case was discussed by a multidisciplinary committee and surgical treatment was decided. Due to the size of the lesion and vascular compromise, we performed a mandibular swing approach, to ensure an adequate surgical field. The mass was fixed to the sternocleidomastoid muscle and the external jugular vein. Dissection started at the CCA caudally and then progressed cranially towards the bifurcation. To attempt negative margins, the ICA and IJV had to be sacrificed. The vagus nerve was encased 360º by the tumor, above and below the carotid bifurcation, so it was resected. The hypoglossal, glossopharyngeal nerves, and the high cervical plexus were preserved.

After the intervention, the patient was extubated and remained in the Intensive Care Unit for the first 48 hours postoperatively. Blood transfusions were not necessary during or after surgery, accounting for an approximate blood loss of 500 ml. 

**Fig 4:**
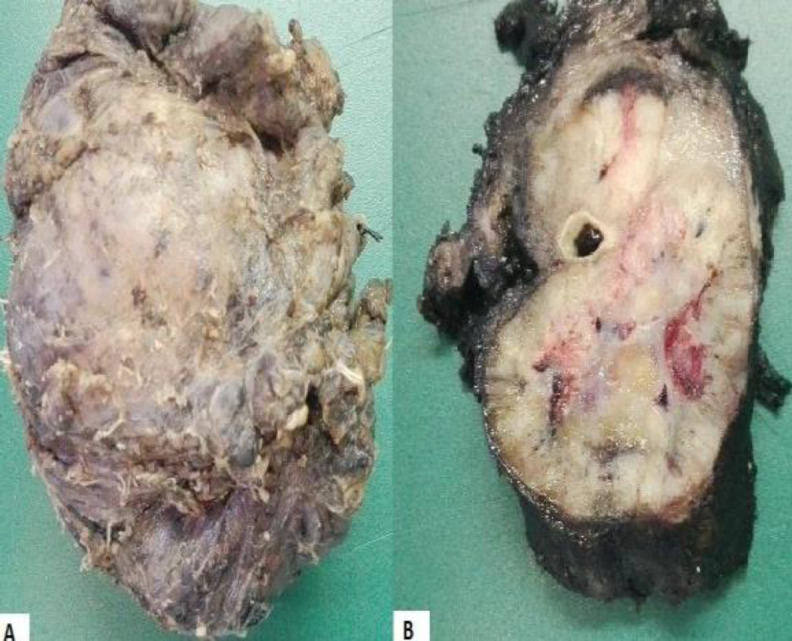
(A-B) Surgical specimen. Carotid body tumor that measures 10 x 8 x 4 cm

**Fig 5:**
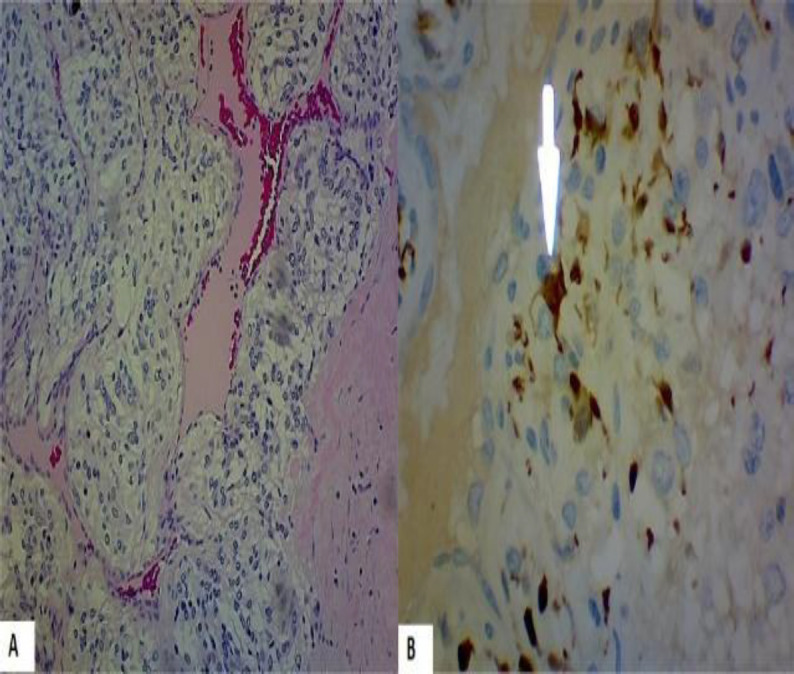
(A) Polygonal or spindle cells arranged in small nests surrounded by sustentacular cells separated by a delicate fibrovascular stroma. Zellballen pattern. Confluent necrosis areas. (H&E 40x) (B) Immunohistochemical staining: S100 positive in the cytoplasm of sustentacular cells. Prolongations of the cytoplasm are observed, hence its name. (40x)

Pathological analysis of the piece revealed a characteristic Zellballen pattern, suggestive of a CBT. Although atypical mitoses were not identified, we found confluent necrosis and infiltration of the capsule among the piece, characteristics highly associated with malignant behavior (Fig4) (Fig5).

With the sacrifice of the vagus nerve, signs of its functional impairment were revealed. The patient presented dysphonia due to ipsilateral vocal cord paralysis, which was subsequently resolved with voice re-education by a speech therapist. She had no subjective difficulty swallowing. 

The patient did not manifest any ischemia-related symptoms.

The aesthetic result was adequate and, in the year following the intervention, there were no signs or symptoms of disease recurrence.

## Discussion

CBTs are an infrequent group of neuroendocrine neoplasms, originating from paraganglionic cells of the carotid body, located at the CCA bifurcation. The incidence of these tumors is around 1-3 cases per million inhabitants per year (7). They typically occur in the fifth decade of life, with almost the same frequency in both genders; although some studies of CBTs occurring at high altitudes have demonstrated a predilection for female patients, as in our case. High altitude has been described as a risk factor by several papers (8-10). 

Case presentations can be sporadic or familial. Familial cases are usually bilateral, synchronous, or metachronous; and may be associated with other paragangliomas (4). In any patient with a CBT, family screening is necessary. 

Part of the sympathetic nervous system, they arise from chromaffin-negative glomus cells of the paraganglia (11). Closely related to pheochromocytomas, they account for only 0.5% of all body tumors; however, they represent about 60-70% of head and neck paragangliomas (12,13). Histologically, CBTs have a characteristic growth pattern consisting of polygonal or spindle cells, that show reactivity with chromogranin and synaptophysin stains, arranged in small nests (characteristic Zellballen pattern) surrounded by sustentacular cells, S100 positive (1,14,15). CBTs are most frequently benign. Malignant transformation and local or distant metastasis approximately represent 10% of cases (16). 

Although the gold standard of diagnosis for malignancy should be based on distant metastasis (17,18), some histological features contribute to differentiating benign tumors from their malignant counterpart; central necrosis, mitotic figures, and infiltration beyond the capsule were reported in several studies (16,19). 

Timely surgery is the optimal treatment and the key to a favorable prognosis. The reasons for performing surgical resection are a) malignant transformation; b) no reliable screening mechanism for monitoring tumor progression; c) eventually they could become symptomatic; d) the risk of vascular or neurological injury is acceptable in appropriate hands (20). The different modalities for the diagnosis of CBTs include ultrasound, computed tomography, and MRI; yet the gold standard for diagnosis is angiography (7,21-23). 

Biopsy to confirm diagnosis should be avoided as it could represent a risk, due to the high vascularity of the lesion. Resection of these tumors requires an accurate preoperative assessment of vessel anatomy and probable consequences of interrupting blood flow. BOT and techniques to maintain vascular flow when a graft is required, have notoriously reduced surgical complications (24). In our case, the angiography revealed the highly vascular nature of the tumor and highlighted the complete compression of the ECA, ICA, and IJV. The BOT showed no manifestations of hypoperfusion and allowed the sacrifice of vital structures, such as the left ICA, without the need for a bypass.

In most cases, separation of the tumor from the carotid artery could be achieved by meticulous dissection in the subadventitial plane, as described by Gordon-Taylor (25). 

If resection of the ICA along with the tumor is necessary, intraluminal vascular shunts or vascular reconstructive techniques using autogenous vein grafts or prosthetic material could be performed (26). Fortunately, in our patient, careful preoperative planning showed us correct opacification of the left vascular territory through the circle of Willis and a complete ligation of the left ICA without the need for reconstruction was performed. However, even with these precautionary measures, vascular reconstruction must be preoperatively considered. During surgery, the cerebral flow was closely measured (27). There is still controversy around the use of preoperative embolization. According to some authors, it could reduce blood flow and decrease tumor size, thereby facilitating tumor excision (28). However, in huge CBTs, as in our case, multiple arteries participate in tumor vascularization, complicating the identification of the feeding vessel. Superselective embolization is challenging and may represent a real risk of particle migration (29,30). Radiotherapy used to treat these patients remains controversial due to several recurrences reported (31,32). 

The indications should, therefore, be limited to only unresectable tumors or non-operable patients. A wait-and-see policy has also been proposed, in some particular cases, since most paragangliomas grow slowly (33). If there is a risk of major postsurgical functional complications, it should be considered. Beyond the selected treatment, careful follow-up of all patients with CBTs is crucial considering the prolonged intervals between initial diagnosis and recurrence, as well as the infrequent but possible late metastasis.

## Conclusion

CBTs are unusual but treatable lesions if resected without metastatic or residual disease. This is why surgery should be performed whenever possible.
